# Annotations

**Published:** 1908-06-13

**Authors:** 


### ANNOTATIONS.

### The Model Prescription.

The discussion and solution of the difficulties connected with the re-dispensing of old prescriptions seem to be more advanced in the United States than in this country, where spasmodic expressions of opinion take so long in translation into active measures. The American Pharmaceutical Association in New York has adopted a series of principles which embody most of the reforms for which urgency is occasionally claimed in Great Britain. Perhaps the most important of these is that contained in declaration 3, which states that the medicine prescribed should be supplied not more than once, if the prescription is marked "Ne repetatur," or "Not to be repeated"; if it contains narcotic or habit-forming drugs; and if asked for by someone known not to be the original patient. It is considered advisable also that the pharmacist should retain the prescription and preserve it for not less than five years, and that he should give a copy of it to the patient unless the prescriber indicates the contrary on the prescription. It is also suggested that besides the name of the patient, the prescription should bear the age whenever it concerns a child, and that whenever an unusually large dose is being ordered it should be underlined and followed by an exclamation mark. It was scarcely, perhaps, to be expected that medical caligraphy should escape attention, for the first recommendation enjoins, amongst other things, that the writing shall be plain and distinct, and that the author's full name shall be appended. In view of the fearful illegibility of some medical prescriptions it cannot be maintained that either of these remarks is out of place, though there may be something to be said in favour of such a degree of contraction or of bad writing as may render the prescription difficult of comprehension to the untrained and yet sufficiently clear to the initiated. We say this not from any desire for an atmosphere of mediæval mystery, but simply because for the patient's own sake it is better that he should not be permitted to know.

### "Preserved" Cream.

In view of the near approach of the strawberry season considerable interest attaches to a recent decision of the High Court on the admixture of boric acid with cream. The Court of Quarter Sessions upheld a magisterial decision by which a grocer was convicted of an offence under the Sale of Food and Drugs Act for selling, in response to a demand for cream, two pots of that delicacy containing 0.313 per cent. of boric acid. The labels bore a statement that "this cream contains a small percentage of boron preservative to retard sourness"; but this was held not to constitute a valid defence. The King's Bench Judges apparently took the view that if "preserved" or "potted" cream had been asked for, there would have been no offence; but that the label would not convey to purchasers very much about the nature of the added ingredient. They therefore upheld the decision of the police magistrate and of the lower Court. The chief argument which weighed with all these authorities in coming to what most folk will certainly think a very sensible decision, was that the percentage of added boric acid, though not injurious to healthy adults, might well be so to children or invalids, who together constitute a very considerable section of the population. In point of fact, the Local Government Board allows the addition of boric acid to milk in the proportion of forty grains per gallon, but this works out at about one-sixth of the quantity added to the defendant's cream. It appeared in evidence, however, that less than 0.3 per cent. of the preservative is useless for cream, and even more is required in very hot weather. The practical upshot of the case is that those of the public who are in good health and like cream at a reasonable price must be content to accept it "preserved" with boric acid, whereas for invalids and babies the pure article can be procured at a rather greater cost. If this comes to be clearly understood, no one will have ground for complaint, and the unfortunate defendant may console himself with the profitless reflection that he is a public benefactor.

### Enteric Fever in Belfast.

The Irish Local Government Board's special commission of inquiry into the alleged undue prevalence of enteric fever in Belfast has revealed several matters which one would hesitate to believe possible in the twentieth century were they not so fully attested by recognised authority. Thus it comes out that no returns of deaths are communicated by the local registrars to the sanitary authorities of their districts, for in Ireland there is no law requiring them so to do. It is impossible for the Belfast Corporation to find out even how many deaths occur in the whole city, let alone in what sections of it the rates of mortality are highest and by what diseases the total is swelled. It was even uncertain whether there was actually any unusual enteric fever morbidity in the city, for the belief was founded, quite justly as it turns out, simply on the general impression of the local medical men. In face of disabilities such as these the prophylaxis of any preventable disease becomes almost impossible, and the strong recommendation that Ireland should be brought under the same Acts as are in force in the rest of the United Kingdom is one against which dissentients will have to produce some very cogent argument. The detailed investigation of the registrar's returns showed that the death-rate from enteric fever has, in fact, been persistently for many years nearly five times that in England and Wales, notwithstanding great improvements in the water supply and in the general sanitation. To cut a long story short, the custom of eating uncooked shell-fish from the sewage-contaminated Lough is established practically beyond all doubt as the cause of the mischief; and as it seems impossible fully to safeguard the waters from such contamination, the drastic remedy is suggested of prohibiting altogether the gathering of shell-fish there for human consumption. It is difficult to see what better course can be adopted, for, in view of the facts elicited, it is obvious that some radical measure is imperative.

